# Transfusion of platelets, but not of red blood cells, is independently associated with nosocomial infections in the critically ill

**DOI:** 10.1186/s13613-016-0173-1

**Published:** 2016-07-19

**Authors:** Leo J. Engele, Marleen Straat, Ingeborg H. M. van Rooijen, Karen M. K. de Vooght, Olaf L. Cremer, Marcus J. Schultz, Lieuwe D. J. Bos, Nicole P. Juffermans, MARS Consortium, Friso M. de Beer, Roosmarijn T. M. van Hooijdonk, Mischa A. Huson, Laura R. A. Schouten, Marcus J. Schultz, Lonneke A. van Vught, Maryse A. Wiewel, Esther Witteveen, Gerie J. Glas, Luuk Wieske, Tom van der Poll, Marc. J. M. Bonten, Jos F. Frencken, Peter M. C. Klein Klouwenberg, David S. Y. Ong

**Affiliations:** Department of Intensive Care Medicine, Academic Medical Center, University of Amsterdam, Meibergdreef 9, 1105 AZ Amsterdam, The Netherlands; Transfusion Laboratory, Academic Medical Center, University of Amsterdam, Amsterdam, The Netherlands; Laboratory of Experimental Intensive Care and Anesthesiology (LEICA), Academic Medical Center, University of Amsterdam, Amsterdam, The Netherlands; Department of Clinical Chemistry and Haematology, University Medical Center Utrecht, Utrecht, The Netherlands; Department of Intensive Care Medicine, University Medical Center Utrecht, Utrecht, The Netherlands

**Keywords:** Nosocomial infection, Critically ill, Red blood cells, Fresh-frozen plasma, Platelets, Transfusion

## Abstract

**Background:**

Red blood cell (RBC) transfusion has been associated with nosocomial infection in the critically ill patients. However, this association may be confounded by length of stay, as prolonged intensive care unit (ICU stay) increases both risk of infection and risk of transfusion. Also, it is not known whether specific blood products have differential risks.

**Methods:**

In this prospective multicentre cohort study, the risk of bacterial infections associated with transfusion products in critically ill (ICU) patients was determined in an integrated statistical model, using Cox proportional hazard analysis to account for attrition bias. In all acutely admitted patients with a length of stay of >48 h between 1 January 2011 and 31 December 2012, the occurrence of nosocomial infections in the ICU was prospectively monitored using CDC criteria.

**Results:**

Of 3502 screened patients, 476 (13.6 %) developed a nosocomial infection. These patients had higher APACHE IV scores, had longer ICU length of stay and were more frequently transfused compared to patients without an infection. Logistic regression showed that RBC transfusion was a risk factor for infection [odds ratio (OR) 1.98, 95 % confidence interval (CI) 1.54–2.55, *p* < 0.001], as well the number of RBC units transfused (OR 1.04, 95 % CI 1.03–1.06, *p* < 0.001). However, these associations disappeared in the Cox proportional hazard analysis. In contrast, we found an association between plasma transfusion and infection [hazard ratio (HR) 1.36, 95 % CI 1.10–1.69, *p* = 0.004] and between platelet transfusion and infection (HR 1.46, 95 % CI 1.18–1.81, *p* < 0.001). However, only platelet transfusion was associated with infection independently from other transfusion products (HR 1.40, 95 % CI 1.03–1.90, *p* = 0.03).

**Conclusions:**

In critically ill patients, transfusion of platelets, but not of RBCs and plasma, is an independent risk factor for acquiring a nosocomial infection.

**Electronic supplementary material:**

The online version of this article (doi:10.1186/s13613-016-0173-1) contains supplementary material, which is available to authorized users.

## Background

In medical and surgical critically ill patient populations, an increased risk of nosocomial infections following red blood cell (RBC) transfusion has repeatedly been demonstrated in observational studies [1–11]. The risk of infection following RBC transfusion has been related to the amount of transfused blood [1, 2, 7] as well as to RBC storage lesion, although studies show conflicting results on the association between storage duration and infection [2, 4–6, 8, 11]. Given that the chance of receiving stored blood increases when the amount of transfusion is higher, it is a challenge to determine whether increased risk of infection following transfusion is due to storage or due to the number of transfused units. Regardless of the mechanism, the association between transfusion and infection can be confounded by length of stay in the intensive care unit (ICU), because both the risk of receiving a (stored) blood transfusion and the risk of developing an infection increase during prolonged ICU stay. This is called attrition bias. Previous studies have not always accounted for this type of bias.

As RBCs are often administered together with plasma and platelets, another challenge may be to dissect whether these other blood products influence the risk of infection. This is relevant, as also platelet transfusions have been associated with post-operative infection in cardiac surgery patients [12] and in critically ill patients recovering from sepsis [13]. These immunosuppressive effects of transfused platelets have been related to alterations in their expression of MHC class I molecules [14]. Also, plasma has been related to infectious complications. An observational study found that plasma increased the risk of infection in critically ill surgical patients, which was not due to concomitant RBC transfusion [15]. Also in cardiac surgery, FFP was associated with nosocomial infection [16].

The purpose of this prospective cohort study was to investigate the risk of nosocomial infections following transfusion in critically ill patients. We hypothesized that the number of blood products was independently associated with an increased risk of nosocomial infection. Cox regression was used to adjust for the effect of attrition bias. An integrated model was used to study the independent effect of the different blood products.

## Methods

### Design and subjects

This study was performed within the framework of the Molecular Diagnosis and Risk stratification of Sepsis (MARS) project [17]; a prospective observational cohort study was performed in the mixed surgical–medical ICU of two university hospitals in the Netherlands, in which all admissions between the period of 1 January 2011 and 31 December 2012 were included, with the exception of cardiac surgery patients. The presence of bacterial nosocomial infection was the primary outcome of this study. The medical ethics committees of the participating study centres gave approval for an opt-out consent method (IRB number 10-056C). Patients with an ICU stay of less than 48 h were excluded.

### Outcomes

All data were gathered prospectively by a team of trained research fellows, using an electronic format. Scoring of nosocomial infection was performed daily based on criteria adapted from the Centre of Disease Control that were published previously [17]. These criteria included the source of infection, the causative pathogen and the plausibility of infection (none, possible, probable and definite). Infections are both post-surgical and non-surgical. Only the first infection episode was included in this study. Detailed definitions of infection are given in Additional file 1. In this study, patients with a possible, probable or definite bacterial infection were included. The overall inter-observer variability for all sites of infection was 89 %, as previously reported [17]. A cleaning algorithm was used, which alarmed the researchers of inconsistency of data, after which a hand check of the data was done. Use of this algorithm resulted in an increase in inter-observer agreement to 85 % for ventilator-associated pneumonia (VAP) [15], which accounted for 26 % of the nosocomial infections in this study.

### Risk factors for infection

Potential confounders were selected based on previous data [13, 18] and included mechanical ventilation, sepsis at ICU admission, Acute Physiology and Chronic Health Evaluation (APACHE) predicted length of stay, admission type, cancer, immunosuppressive medication, immunosuppressive condition and trauma.

 As part of standard care, patients with an anticipated ICU stay of more than 2 days received either selective digestive tract decontamination (SDD, consisting of a non-absorbable solution of tobramycin, colistin and amphotericin B administered in the buccal cavity and as a suspension through the nasogastric tube, given for the length of ICU stay, combined with cefotaxime given for 4 days), or selective oropharyngeal decontamination (SOD, consisting only of administration of the non-absorbable solution in the oropharynx). Transfusion data (date of infusion, type and amount of blood product) of all blood transfusions which were given during ICU stay and 1 week prior to ICU admission were prospectively registered in a patient digital monitoring system. The storage time of blood products was obtained from the hospital blood transfusion service. In patients who developed a nosocomial infection, only transfusions given before the onset of infection were evaluated. Per blood product, a qualitative variable (transfusion yes or no) and a quantitative variable (amount of product in units) were generated. Stratification for RBC storage time was done dichotomous (>14 days). RBCs were stored in SAGM. Platelet products were pooled from 5 donors and stored in plasma. All blood products were prestorage leuko-reduced. Fresh-frozen plasma (FFP) was prepared from male donors only.

Transfusion protocols were similar in both institutions, holding that one RBC unit is transfused to correct for anaemia at a general haemoglobin trigger of 7 g/dL. A unit of pooled platelets from 5 donors is transfused prophylactically at a platelet count of 10 × 10^9^/L or of 50 × 10^9^/L in case of use of antiplatelet medication. FFPs, RBCs and platelets are liberally transfused at a 1:1:1 ratio (i.e. five units erythrocytes/five units FFP/one 5-donor unit platelets) during haemorrhage.

### Statistical analysis

We analysed the association between nosocomial infection and RBCs, FFPs or platelets separately. We corrected for potential confounders, the risk of receiving a transfusion (using propensity scores) and attrition bias (ICU observation period) using a five-step approach described below and visualized in Additional file 2: Figure S1. First, we evaluated which features of the blood transfusions (qualitative; transfusion yes/no; quantitative; total number; and/or the age of blood products) were associated with infection (details in Additional file 2: Figure S1). A logistic regression model with nosocomial infection as the dependent variable was used, and non-informative variables were removed using backward selection by Akaike information criterion (AIC). Second, the effect of transfusion was corrected for confounding effects (see Additional file 2). As in step one, we used backward selection to identify confounders from a group of predefined variables (see *risk factors for infection*). Third, propensity scores (for explanation, see Additional file 2) were calculated to estimate the influence of the a priori likelihood of receiving a blood transfusion on the development of a nosocomial infection using a logistic regression model with transfusion as dependent variable. The propensity score was added as a co-variate in the logistic regression model with nosocomial infection as dependent variable. Fourth, we adjusted for the effect of attrition bias (ICU observation period) by means of Cox regression. Cox regression was performed with the same independent variables (transfusion features, confounders and propensity score) as in step 3. A Kaplan–Meier curve was used to visualize the hazard of nosocomial infection per transfusion product. A sensitivity analysis with a mixed-effect Cox proportional hazards model was performed to evaluate the differences in effect size between the two hospitals. The impact of missing data was studied by repeating the previous analyses after multivariate imputation by chained equations and comparing the proportional hazard between the model with and without missing data. Finally, we studied the independent association of the three transfusion products with nosocomial infection by inclusion of all variables that were selected in the three previous models into one Cox proportional hazard model. Only features of transfusion that remained significantly associated with infection in this step were considered to be independently associated with nosocomial infection.

Differences between the groups were compared using the Student’s *T* test (normally distributed variables) or Mann–Whitney test (non-normally distributed variables) for continuous variables. The Chi-square test was used for categorical variables. Data were summarized using the mean and standard deviation (SD) for normally distributed variables and the median and inter-quartile range (IQR) for non-normally distributed variables. Categorical variables were expressed in absolute numbers and percentages. All analyses were performed in R statistics using the RStudio interface (www.rstudio.com, version 0.98.501). *p* values below 0.05 were considered significant.

## Results

Of 3502 ICU patients, 37 % were transfused with any blood product. Patients were most frequently transfused on the first day of ICU admission. With regard to the total cohort, 476 patients (13.6 %) developed a nosocomial bacterial infection during their ICU stay. The median time to infection was 6 days (IQR 3–10). Of all infections, 56 % occurred early (0–6 days) and 44 % occurred late (>6 days). With regard to the probability of infection, 28 % of infected patients were scored as having a definite infection, 17 % had a probable infection and 55 % had a possible infection (for definitions, see Additional file 2). Characteristics summarized for the patients with infection and without an infection are listed in Table 1. The median ICU stay of patients who developed a nosocomial infection was longer compared to patients who did not develop an infection [14 (IQR 8–26) vs. 4 (IQR 2–7) days, respectively]. Furthermore, patients with an infection had a significant higher APACHE IV score compared to patients without an infection. The most frequent site of infection was the respiratory tract (51 %). Gram-positive pathogens were the most frequent organisms found in nosocomial infections (Table 2).

**Table 1:** Baseline characteristics

	Overall (*N* = 3502)	Infected (*N* = 476)	Non-infected (*N* = 3026)	*p* value
Mean age (years ± SD)	59.1 ± 16.2	59.6 ± 15.9	59.0 ± 16.3	<0.001
Gender, male [*n* (%)]	2129 (61)	310 (65)	1819 (60)	0.04
APACHE IV score, median (IQR)	72 (54–92)	79 (63–100)	71 (53–91)	<0.001
Readmission [*n* (%)]	552 (16)	83 (17)	469 (15)	0.28
Admission type				0.01
Medical [*n* (%)]	2040 (58)	252 (53)	1788 (59)	
Surgical elective [*n* (%)]	665 (19)	91 (19)	574 (19)	
Surgical emergency [*n* (%)]	795 (23)	133 (28)	662 (22)	
Any transfusion [*n* (%)]	1304 (37)	288 (61)	1016 (34)	<0.001
Solid malignancy [*n* (%)]	454 (13)	54 (11)	400 (13)	0.26
Haematologic malignancy [*n* (%)]	137 (4)	20 (4)	117 (4)	0.73
Immunosuppressive condition (*n*)	392 (11)	56 (12)	336 (11)	0.67
HIV infection [*n* (%)]	41 (1)	6 (1)	35 (1)	0.84
Immunosuppressive medication [*n* (%)]	367 (10)	54 (11)	313 (10)	0.51
Trauma [*n* (%)]	280 (8)	51 (11)	229 (8)	0.02
Mechanical ventilation [*n* (%)]	3127 (89)	455 (96)	2672 (88)	<0.001
Sepsis [*n* (%)]	1544 (44)	189 (40)	1355 (45)	0.04

*APACHE* Acute Physiology and Chronic Health Evaluation, *HIV* human immunodeficiency virus, *IQR* inter-quartile range, *SD* standard deviation

**Table 2:** Site of infection and causative organisms in infected patients

	*n* = 476 No. (%)
Infection site	
Respiratory tract (HAP, VAP, empyema)	242 (42.0)
Intra-abdominal (peritonitis, biliary tract infection, abscess)	42 (8.8)
Cardiovascular (BSI, endocarditis, mediastinitis)	88 (18.5)
Soft tissue (erysipelas, phlebitis, abscess, decubitus infection)	12 (2.5)
Post-operative wound (superficial, deep)	11 (2.3)
Renal/urinary tract (urosepsis, upper urinary tract infection)	9 (1.9)
Central nervous system (brain abscess, meningitis)	44 (9.2)
Others (bones/joints, reproductive system, oral infections)	14 (2.9)
Unknown	10 (2.1)
Microorganisms	
Gram-positive	148 (31.0)
Staphylococcus aureus	38 (8.0)
Staphylococcus epidermis	26 (5.5)
Streptococcus species	10 (2.1)
Enterococcus species	65 (13.7)
Other	9 (1.9)
Gram-negative	139 (28.6)
Enterobacteriaceae species	68 (14.3)
Haemophilus influenza	18 (3.8)
Pseudomonas species	40 (8.4)
Other	13 (2.7)
Other	10 (2.1)
Anaerobes	6 (1.3)
Unknown	173 (36.3)

*HAP* hospital-acquired pneumonia, *VAP* ventilator-acquired pneumonia, *IAI* intra-abdominal infection, *BSI* blood stream infection

Overall, RBCs were given in 35 % of admitted patients, FFP in 17 % and platelets in 18 % of patients. Most patients (46 %) were transfused on the day of ICU admission. Transfusion characteristics are listed in Table 3 and Additional file 2: Table S1. Patients receiving transfusion had a higher APACHE IV score than non-transfused patients. There were no apparent differences between patients receiving RBCs and those receiving other blood products. The patients with infection more often received transfusion compared to the patients without infection, including more units stored for prolonged time.

**Table 3:** Transfusion characteristics

Patients	Overall (*n* = 3502)	Infected (*N* = 476)	Non-infected (*N* = 3026)	*p* value
RBC [*n* (%)]	1235 (35)	272 (57)	963 (32)	<0.001
RBC > 14 days [*n* (%)]	1053 (30)	224 (47)	829 (27)	<0.001
FFP [*n* (%)]	602 (17)	159 (33)	443 (15)	<0.001
Platelets [*n* (%)]	621 (18)	157 (33)	464 (15)	<0.001
Time between (first) transfusion and infection (days)				
RBC [median (IQR)]		6 (3–10)		
FFP [median (IQR)]		6.5 (4–10)		
Platelets [median (IQR)]		7 (4–10)		

Blood products in transfused patients	Overall (*n* = 1304)	Infected (*n* = 288)	Non-infected (*N* = 1016)	*p* value
RBC				
No. of units [median (IQR)]	4 (2–8)	5 (2–11)	4 (2–7)	<0.001
No. of units >14 days [median (IQR)]	3 (1–6)	4 (1–8)	2 (1–6)	<0.001
FFP				
No. of units [median (IQR)]	0 (0–0)	0 (0–3)	0 (0–0)	<0.001
Platelets				
No. of units [median (IQR)]	0 (0–0)	0 (0–2)	0 (0–0)	<0.001

*RBC* red blood cells, *FFP* fresh-frozen plasma, *IQR* inter-quartile range

*Red blood cells* The median storage time of RBC units in this study was 20 days (IQR 16–23). Forty-seven percentage of the patients with infection received at least one RBC unit older than 14 days and 34 % received at least one RBC unit older than 21 days. The patients with infection received a larger amount of RBC units and a larger amount of older RBC units compared to patients without infection (Table 3).

Logistic regression identified both transfusion of RBC as a qualitative, dichotomous variable (yes or no) and amount of transfusion as independent predictors for nosocomial infection. This association remained significant after adjustment for potential confounders and exposure bias to transfusion (the risk of receiving a transfusion). However, the proportional hazard model showed no significant association between nosocomial infection and both RBC transfusion and amount of transfusion (Table 4). We did not observe an independent association between the storage time (RBC units >14 and RBC units >21 days) of RBCs and onset of nosocomial infection (Additional file 2: Table S2).

**Table 4:** Analysis of association between transfusion products and nosocomial infection in separate models

Blood product	Logistic regression			Cox regression		
	Odds ratio	95 % CI	*p* value	Hazard	95 % CI	*p* value
Red blood cells						
RBC transfusion^a^	1.977	1.535–2.547	<0.001	1.143	0.906–1.442	0.259
RBC units^a^	1.044	1.026–1.063	<0.001	1.014	1.000–1.028	0.053
Fresh-frozen plasma						
FFP transfusion^b^	2.510	1.978–3.186	<0.001	1.362	1.101–1.685	0.004
Platelets						
Platelet transfusion^c^	2.530	1.998–3.205	<0.001	1.463	1.184–1.806	<0.001

*FFP* fresh-frozen plasma, *RBC* red blood cells

^a^Corrected for exposure bias (for RBC transfusion) summarized in propensity score including trauma, malignancy, admission type, APACHE IV score and sepsis. Also corrected for confounders including APACHE predicted length of stay and mechanical ventilation

^b^Corrected for exposure bias (for FFP transfusion) summarized in propensity score including admission type, APACHE IV score and sepsis. Also corrected for confounders including APACHE predicted length of stay and mechanical ventilation

^c^Corrected for exposure bias (for platelet transfusion) summarized in propensity score including admission type, malignancy, APACHE IV score and sepsis. Also corrected for confounders including APACHE predicted length of stay and mechanical ventilation

*FFP* The logistic regression model identified FFP transfusion as a risk factor for nosocomial infection. This association persisted in the proportional hazard model (Table 4). We found no association between the amount of FFP transfusion and nosocomial infection.

*Platelets* In the sub-analysis with platelets, both logistic regression model and Cox regression showed that transfusion of platelets was an independent risk factor for the onset of nosocomial infection. A hazard of 1.40 means that a subject’s hazard at any given time is increased relative to the baseline hazard with 40 %, while the baseline hazard may vary.

*Combined analysis* In the combined analyses for the three different types of blood transfusions, only platelet transfusion remained as an independent risk factor for the development of nosocomial infection, i.e. independent from the effect of RBCs and plasma (Table 5).

**Table 5:** Analysis of independent transfusion risk factors for nosocomial infection in a combined model

Blood product	Cox regression		
	Hazard ratio	95 % CI	*p* value
Red blood cells			
RBC transfusion^a^	1.088	0.843–1.403	0.516
RBC units^a^	0.995	0.978–1.012	0.559
Fresh-frozen plasma			
FFP transfusion^a^	1.100	0.808–1.499	0.545
Platelets			
Platelet transfusion^a^	1.401	1.033–1.901	0.030

^a^Corrected for exposure bias summarized in propensity scores of the individual transfusion products and corrected for confounders including APACHE predicted length of stay and mechanical ventilation

*Sensitivity analysis* To evaluate the differences in effect size between the two hospitals, mixed-effect Cox regressions were done, which showed similar effect sizes, suggesting stable models. The Cox models with imputed missing data showed a significant effect for FFP transfusion and platelets transfusion, suggesting that missing data did not influence results. The influence of pre-ICU transfusions was also investigated, as these may influence risk of infection while on ICU. Additional file 2: Tables S5 and S6 show that the different outcomes were not sensitive to exclusion of the pre-ICU transfusion period. To correct for disease severity occurring later during ICU admission, the model was re-run with SOFA score on day 3. The HR of infection following platelet transfusion remained significant in this model.

## Discussion

This study did not demonstrate an association between RBC transfusion and nosocomial infection in ICU patients after adjusting for ICU length of stay. However, we found that transfusion of platelets was independently associated with nosocomial infection.

Differences between our results and previous studies [1, 2, 7, 19], which demonstrated an association between RBC transfusion and infection, may be explained by the use of different statistical analysis methods. Our results are based on both multivariate logistic regression and Cox regression, whereas in previous studies, Cox regression is often not used, thereby not accounting for exposure time. Correcting for exposure time in this study may have negated the finding of an association of RBC and infection, suggesting that both transfusion and infection are conditions that occur as a result of ICU stay but are not causally related. Alternatively, differences may be explained by using different models. Of note, plasma or platelets are mostly transfused concomitantly with RBCs. In this study, risks of blood products were analysed in one model instead of analysing effects of products separately, which is more comparable to real life. Explanations other than different statistical methods include differences in preparation methods and use of storage solutions. Also, differences in case mix may explain different findings, as most studies on transfusion and infection were done in trauma patients.

We did not find an association between RBC storage time and nosocomial infection. This is in contrast to other studies [3, 6], but in line with recent large trials in which ICU patients or cardiac surgery patients were randomized to fresh or stored blood [4, 20].

We found that the hazard ratio of infection was higher in patients receiving platelets, an effect that was independent of the other transfusion products. Besides their haemostatic potential, platelets are recognized to play a role in innate immunity, including activation and homing of leucocytes and production of pro-inflammatory cytokines [21]. In line with this, transfusion of platelets can induce transfusion-associated immunomodulation in a mouse model, associated with the expression of platelets MHC class I antigens [14]. Clinical studies also underline an association between platelet transfusion and infection [12, 13, 16], although not all studies are consistent [22]. Given that RBCs were not associated with occurrence of infection in the large group of patients that received only RBCs in this study, it is unlikely that RBCs contributed to the risk of infections when administered together with platelets. Alternatively, one may argue that patients receiving platelets are mostly haematologic patients who are at risk of nosocomial infections. However, patient characteristics did not differ between those receiving platelets and those receiving other blood products. Thereby, this study suggests that platelet transfusions are the most important blood products associated with increased risk of infection in the critically ill. The mechanism by which platelets may reduce host response to infection remains to be determined.

Does this finding have clinical relevance? Arguably, if indicated, platelet transfusions cannot be omitted. However, clear indications for platelet transfusion in ICU patients are lacking. Prophylactic platelet transfusion prevents bleeding in haematology patients [23], but it is not known at which count platelets should be transfused in ICU patients to prevent bleeding. Protocols at both centres pragmatically suggest a count of 10,000 as a trigger for transfusion in ICU patients and a trigger of 50,000 when the patient needs treatment with anticoagulant medication. These suggestions are, however, not substantiated with data. The lack of data on clear indications, together with the suggestion of possible harm in this study, calls for studies on the risk–benefit of platelet transfusion in the critically ill.

This study has some important limitations. Causality between platelet transfusion and infection cannot be established in this study. Even after adjustment for illness severity, platelet transfusion may still be a surrogate marker for sicker patients. Causality can only be determined in an RCT. Also, half of the patients complied to the definition of possible infection, which may have induced misclassification, including those with a post-transfusion reaction. Statistical modelling using only patients with definite infection was not possible in this study due to a lack of power, even in this relatively large cohort. However, even if this analysis was possible, the use of a very strict definition (e.g. culture positive sepsis) may lead to selection bias, as the amount of culture negative patients is known to be more than one-third of all sepsis patients [24]. Also, the use of clear definitions in this study limited subjectivity of classification, as suggested by a low inter-observer variation in this study. Still, the diagnosis of infection remains an uncertainty in this observational study, requiring follow-up studies to affirm our findings.

Other limitations of this study include preparation method. Our results may not be translated to other settings where these methods differ. Furthermore, among ICU centres there is variability in ICU-specific factors, which can lead to heterogeneity in nosocomial infection hazard rates. Of these, the use of SDD may be the most important. Whether the results in this paper can be extrapolated to centres that do not use SDD remains to be determined. Lastly, although we accounted for exposure bias that is a well-known confounder in studies investigating risk factors for nosocomial infection, standard Cox regression analysis is limited in correcting for time-dependent bias and competing risks. However, this study also has strong aspects. We investigated the association between transfusion products and nosocomial infection with a prospective study design, using a structured evaluation of all infectious events, to minimize the potential sources of bias and confounding. In addition, our study is characterized by a large sample size. Furthermore, the independent effect on nosocomial infections was studied for each type of transfusion product separately.

In conclusion, we investigated the association between different transfusion products and the onset of nosocomial infection in the critically ill patients. We suggest that transfusion of platelets, but not of RBCs, is associated with infection. Further studies on the mechanisms of this association and on possible interventions which may modulate this risk are warranted.

## Additional files

10.1186/s13613-016-0173-1 Definitions of infection.
10.1186/s13613-016-0173-1 Supplemental statistical methods. Table S1.1 Comparison of the combination of blood transfusion products in all transfused patients. Table S1.2 2 Comparison of the combination of blood transfusion products in transfused patients with an infection. Table S1.3 Patient characteristics according to transfusion product. Tables S2. Models for RBC transfusion. Tables S3. Models of FFP transfusion. Table S4. Models for PLT transfusion. Table S5. Logistic and cox regression model for each type of transfusion separately. Table S6 Analysis of independent transfusion risk factors for nosocomial infection. Table S7. Analysis of independent transfusion risk factors for nosocomial Infection with SOFA score on day 3.
