# Supplementary material for: Transfusion of platelets, but not of red blood cells, is independently associated with nosocomial infections in the critically ill
Source: Ann Intensive Care. 2016 Jul 19;6:67. doi: 10.1186/s13613-016-0173-1 (PMC4951387; doi:10.1186/s13613-016-0173-1)
Supplement: Supplementary file 1 — 10.1186/s13613-016-0173-1 Definitions of infection. [file 13613_2016_173_MOESM1_ESM.docx]

Online supplemental file: definitions of infection

Transfusion of fresh frozen plasma and platelets, but not of red blood cells, is associated with nosocomial bacterial infections in the critically ill

Leo J. Engele, BSc^1^, Marleen Straat, MD^1^, Ingeborg H.M. van Rooijen^2^, Karen M.K. de Vooght, PharmD, PhD^4^, Olaf L. Cremer, MD, PhD^5^, Marcus J. Schultz, Lieuwe D.J. Bos, PhD^1^  and Nicole P. Juffermans, MD, PhD^1,3^ ; on behalf of the MARS ConsortiumOnline Supplement

Respiratory tract .......................................................................................................................... 2

Hospital Acquired Pneumonia

Ventilator‐Associated Pneumonia

Lung abscess and empyema

Sinusitis

Cardiovascular ...............................................................................................................................5

Bloodstream infection (BSI): Primary BSI

Bloodstream infection (BSI): Secondary BSI (CRBI‐ catheter related bloodstream infection)

Endocarditis

Mediastinitis

Central nervous system ..................................................................................................................8

Intracranial infections: abscess

Primary meningitis and/or ventriculitis, encephalitis

Secundairy meningitis

Urinary tract ............................................................................................................................... .10

Urosepsis in noncatheterized patients

Urosepsis in catheterized patients

Upper urinary tract infection

Skin and soft tissue ........................................................................................................................12

Skin infection, erysipelas, phlebitis

Surgical site infections: Superficial wounds

Surgical site infections: Deep wounds

Soft tissue infections

Decubitus infections

Intra‐abdominal infection/abscess (IAI)...........................................................................................15

Pancreatic infection, infected necrotizing pancreatitis

Biliary tract infection

Primary peritonitis (spontaneous bacterial peritonitis)

Secondary peritonitis

Tertiary peritonitis

Peritoneal dialysis‐related peritonitis

Gastroenteritis

Infections of reproductive system……………………………………………………………………………………………..18

Osteomyelitis ............................................................................................................................... 18

Infections of mouth, tongue or gums .............................................................................................19

CPIS ............................................................................................................................... .................19

**Respiratory tract**

| **Hospital Acquired Pneumonia** | |
| --- | --- |
| Clinical  setting | Patients with respiratory symptoms that started more than 48 hours after hospital or nursing  home admission, but without mechanical ventilation (or onset of pneumonia within 48 hours after start of mechanical ventilation) |
| Possible | 1. Abnormal chest radiograph of uncertain cause  **and** low clinical suspicion of pneumonia with at least one of the following symptoms/signs:  a) cough  b) new onset of purulent sputum or change in character of sputum  c) fever or hypothermia  d) leukocytosis  e) elevated CRP (>30 mg/l)  f) hypoxemia (pO2<60 mmHg) |
| Probable | 1. Evident new or progressive radiographic infiltrate, consolidation, cavitation, or pleural effusion  **and** high clinical suspicion of pneumonia with at least two of the criteria at possible  **and** one or more of the following:  a) isolation of an etiologic agent from a specimen obtained by transtracheal aspirate, bronchial brushing, or biopsy  b) likely/possible respiratory pathogen in concentrations below threshold (104 in BAL and  103 in protected specimen brush) using quantitative cultures of a lower respiratory  tract sample (endotracheal aspirate, BAL, or protected specimen brush) |
| Definite | 1. Evident new or progressive radiographic infiltrate, consolidation, cavitation, or pleural  effusion  **and** high clinical suspicion of pneumonia with at least two of criteria at probable  **and** at least one of the following:  a) likely/possible respiratory pathogen cultured from blood;  b) likely/possible respiratory pathogen in concentrations above threshold (104 in BAL and  103 in protected specimen brush) using quantitative cultures of a lower respiratory  tract sample  c) isolation of virus from or detection of viral antigen in respiratory secretions  d) diagnostic single antibody titer (IgM) or fourfold increase in paired sera (IgG) for  pathogen  e) histopathologic evidence of pneumonia |
| Comment | • Sputum cultures are not useful in the diagnosis of pneumonia but may help identify the etiologic agent and provide antimicrobial susceptibility data |

| **Ventilator‐Associated Pneumonia** | |
| --- | --- |
| Clinical  setting | Pneumonia in patients who had a device to assist or control respiration continuously through a  tracheostomy or by endotracheal intubation within the 48‐hour period before the onset of  infection, inclusive of the weaning period |
| Possible | 1. Abnormal chest radiograph of uncertain cause  **and** high clinical suspicion of pneumonia with CPIS > 6  **and** isolation of an etiologic agent from a specimen obtained by transtracheal aspirate or  bronchial brushing |
| Probable | 1. Evident new or progressive radiographic infiltrate, consolidation, cavitation, or pleural effusion  **and** high clinical suspicion of pneumonia with CPIS > 6  **and** at least one of the following:  a) likely/possible respiratory pathogen in concentrations above threshold (104 in BAL and  103 in protected specimen brush) using quantitative cultures of a lower respiratory  tract sample  b) likely/possible respiratory pathogen cultured from blood and lower respiratory tract sample |
| Definite | 1. Evident new or progressive radiographic infiltrate, consolidation, cavitation, or pleural  effusion  **and** high clinical suspicion of pneumonia with CPIS > 6  **and** at least one of the following:  a) histopathologic evidence of pneumonia  b) radiographic evidence of lung abscess or pleural empyema and isolation of an etiologic  agent from a specimen |

| **Lung abscess and empyema** | |
| --- | --- |
| Clinical  setting | Patients presentating with respiratory symptoms and clinical or radiographic evidence of abscess |
| Possible | 1. Low clinical suspicion, with at least one of the following signs or symptoms with no other  recognized cause:  a) fever  b) cough, sputum production  **and** low suspicion for abscess on radiographic examination |
| Probable | 1. See criteria at possible  **and** high suspicion for abscess on radiographic examination |
| Definite | 1. See criteria at probable  **and** drainage of pus from suspected lung abscess or empyema by puncture or surgical  operation. The pus has to be clinically evident or confirmed with biochemical or histopathologic procedures.  **and** confirmed etiologic agent visible in gram staining or pathogen isolated from pus culture |

| **Sinusitis** | |
| --- | --- |
| Possible | 1. Patient with clinical suspicion for sinusitis with at least one of the following signs or symptoms with no other recognized cause:  a) fever (>38°C)  b) leukocytosis |
| Probable | 1. See criteria at possible  **and** at least one of the following criteria:  a) positive transillumination with air‐fluid level;  b) radiologically suspected for sinusitis (CT, ultrasound) |
| Definite | 1. See criteria at probable  **and**  a) positive culture (>1000 colonies/ml) of purulent discharge from sinus cavity plus > 5  PMN per oil immersion field  b) in case antibiotics are prescribed: > 5 PMN per oil immersion field |

**Cardiovascular**

| **Bloodstream infection (BSI): Primary BSI** | |
| --- | --- |
| Clinical  setting | Bloodstream infection in a patient without an evident focus |
| Possible | N.A. |
| Probable | N.A. |
| Definite | 1. At least one of the following:  a) patient has a recognized pathogen (defined as a microorganism not usually regarded as a common skin contaminant, i.e., diphtheroids, Bacillus species, Propionibacterium species, coagulase‐negative staphylococci, or micrococci) cultured from one or more blood cultures  b) a common skin contaminant (e.g., diphtheroids, Bacillus species, Propionibacterium  species, coagulase‐negative staphylococci, or micrococci) cultured from two or more blood cultures drawn on separate occasions (including one drawn by venipuncture)  **and** the organism cultured from blood is not related to an infection at another site, including intravascular‐access devices |

| **Bloodstream infection (BSI): Secondary BSI (CRBI‐ catheter related bloodstream infection)** | |
| --- | --- |
| Clinical  setting | Bloodstream infection in a patient with one or more intravascular‐access devices for more than  72 hours |
| Possible | 1. Negative blood culture: culture of blood obtained by venapuncture or other catheter line is  negative  **and** suspected catheter line with one or more of the following criteria:  • In case catheter is removed:  a) catheter tip is positive  b) culture of catheter not performed because fever disappears within 48 hours after  catheter line removal  • in case of a catheter is present:  a) recovery of fever within 48 hours after start of antibiotic treatment  **and** confirmed absence of other possible sources with same pathogen |
| Probable | 1. Clinical signs of infection with at least one of the following criteria:  a) fever (>38C)  b) chills  c) hypotension (systolic pressure <100 mmHg) or need for vasopression  **and** positive or unknown blood culture: culture of peripheral blood (venapuncture) or blood  obtained from other catheter line is positive, or no culture  **and** suspected catheter line with at least one of the following criteria:  • in case catheter is removed:  a) catheter tip is positive for same pathogen that was recovered from blood culture (or  positive for common skin contaminant if no paired blood culture);  b) culture of catheter not performed because fever disappears within 48 hours after catheter line removal  • in case catheter is present:  a) recovery of fever within 48 hours after start of antibiotic treatment  **and** confirmed absence of other sources with same pathogen |
| Definite | 1. Clinical signs of infection (see at probable)  **and** positive blood culture: culture of peripheral blood (venapuncture) or blood obtained from other catheter line is positive  **and** positive catheter tip culture with same pathogen (i.e., catheter colonization), whereby the same microorganism (species and antibiogram) is isolated from the catheter segment **and** peripheral blood  and confirmed absence of other sources with same pathogen |

| **Endocarditis** | |
| --- | --- |
| Clinical  setting | Patients presenting with SIRS/sepsis without an evident clinical focus, or with persistent  SIRS/sepsis despite adequate therapy for any suspected alternative source |
| Modified  Duke criteria | Major criteria include:  a) positive blood culture with typical IE microorganism, defined as one of the following:  i. typical microorganism consistent with IE from 2 separate blood cultures, as noted  below: Viridans‐group streptococci, or Streptococcus bovis including nutritional variant strains, or HACEK group, or Staphylococcus aureus, or Community‐acquired Enterococci, in the absence of a primary focus  ii. microorganisms consistent with IE from persistently positive blood cultures defined  as:  a. two positive cultures of blood samples drawn >12 hours apart, or  b. all of 3 or a majority of 4 separate cultures of blood (with first and last sample  drawn 1 hour apart)  c. Coxiella burnetii detected by at least one positive blood culture or antiphase I  IgG antibody titer >1:800  b) evidence of endocardial involvement with positive echocardiogram defined as  c) oscillating intracardiac mass on valve or supporting structures, in the path of regurgitant jets, or on implanted material in the absence of an alternative anatomic explanation  d) abscess  e) new partial dehiscence of prosthetic valve or new valvular regurgitation (worsening or  changing of preexisting murmur not sufficient) Minor criteria include:  a) predisposing factor: known cardiac lesion, recreational drug injection  b) fever >38°C  c) evidence of embolism: arterial emboli, pulmonary infarcts, Janeway lesions, conjunctival  hemorrhage  d) immunological problems: glomerulonephritis, Osler's nodes  e) positive blood culture (that doesn't meet a major criterion) or serologic evidence of infection with organism consistent with IE but not satisfying major criterion |
| Rejected  (none) | 1. One of the following criteria  a) evidence for alternative diagnosis  b) symptoms disappearing within 4 days after start of antibiotic treatment  c) no pathological evidence for endocarditis form surgery or autopsy, with less than four  days of antibiotic treatment  d) does not meet the criteria for possible endocarditis, see below |
| Possible | 1. Two of the minor criteria without any other apparent cause |
| Probable | 1. Three minor criteria without any other apparent cause  2. One major and one minor criterion |
| Definite | 1. Clinical criteria with at least 2 major criteria, 1 major and three minor criteria, or 5 minor  criteria  2. Histopathologic evidence: pathogen confirmed in vegetation or abscess |

| **Mediastinitis** | |
| --- | --- |
| Clinical  setting | Patients after cardiothoracic surgery or other causes of an open chest presenting with symptoms  suggestive of an infection of the mediastinum |
| Possible | 1. At least two of the following signs or symptoms in patients after cardiothoracic surgery or  other causes of an open chest:  a) fever (>38°C)  b) chest pain  c) sternal instability |
| Probable | 1. See criteria ‘possible’  **and** at least one of the following:  a) organisms cultured from blood  b) mediastinal widening on x‐ray |
| Definite | 1. See criteria ‘possible’  **and** at least one of the following:  a) purulent discharge from mediastinal area;  b) organisms cultured from discharge from mediastinal area  2. Evidence of mediastinitis seen during a surgical operation or histopathologic examination  3. Organisms cultured from mediastinal tissue or fluid obtained during a surgical operation or needle aspiration |

**Central nervous system**

| **Intracranial infections: abscess** | |
| --- | --- |
| Possible | 1. At least two of the following signs or symptoms with no other recognized cause:  a) headache  b) fever (>38°C)  c) localizing neurologic signs  d) changing level of consciousness  e) confusion |
| Probable | 1. See criteria ‘possible’  **and** at least one of the following:  a) Organisms seen on microscopic examination of brain or abscess tissue obtained by  needle aspiration or by biopsy during a surgical operation or autopsy  b) Radiographic evidence of infection, e.g. abnormal findings on ultrasound, CT scan,  magnetic resonance imaging (MRI), or arteriogram |
| Definite | 1. Organisms cultured from brain tissue or dura  2. Abscess or evidence of intracranial infection seen during a surgical operation or histopathologic examination |

| **Primary meningitis and/or ventriculitis, encephalitis** | |
| --- | --- |
| Possible | 1. At least two of the following signs or symptoms with no other recognized cause:  a) fever (>38°C)  b) headache c) stiff neck  d) meningeal signs  e) cranial nerve signs  f) changing level of consciousness  g) petechia seen during meningococcemia |
| Probable | 1. See criteria at possible  **and** at least one of the following:  a) positive antigen test of cerebrospinal fluid (CSF), blood or urine b) organisms cultured from blood  c) uncertain abnormal CSF findings  d) In case no CSF is obtained due reasons other than clinical motives |
| Definite | 1. Organisms cultured from CSF (if low numbers of skin contaminants, then take chemical and clinical signs into account)  2. See criteria at possible  **and** at least one of the following:  a) Increased white cells, elevated protein, and/or decreased glucose in CSF (if bleedy LP  then positive is when leukocytes:erythrocytes ratio is >1:100; if not bleedy then positive when leukocytes > 100x106/L)  b) organisms seen on Gram stain of CSF  c) positive PCR on viral etiologic agents |

| **Secundairy meningitis** | |
| --- | --- |
| Clinical  setting | Patients presenting with symptoms of meningitis up to 1 year after neurotrauma, neurosurgery,  ENT‐surgery, external ventricular drain, external lumbar drain, or ventriculo‐peritoneal drain if  there is a suspected infection in the peritoneal part |
| Possible | 1. At least two of the following signs or symptoms with no other recognized cause:  a) fever (>38°C)  b) headache  c) stiff neck  d) meningeal signs  e) cranial nerve signs  f) changing level of consciousness |
| Probable | 1. See criteria at possible  **and** at least one of the following:  a) positive urine antigen test (pneumococcal)  b) organisms cultured from blood |
| Definite | 1. Organisms cultured from CSF (if low numbers of skin contaminants, then take chemical and  clinical signs into account)  2. See criteria at possible  **and** increased elevated protein, and/or decreased glucose in CSF (if bleedy LP then positive is  when leukocytes:erythrocytes ratio is >1:100; if not bleedy then positive when leukocytes >  100x106/L)  **and** organisms seen on Gram stain of CSF |

**Urinary tract**

| **Urosepsis in noncatheterized patients** | |
| --- | --- |
| Possible | 1. At least two of the following signs or symptoms with no other recognized cause  a) fever (>38 °C)  b) urgency  c) frequency d) dysuria  e) pyuria |
| Probable | 1. See criteria at ‘possible’  **and** at least one of the following  a) positive dipstick for leukocyte esterase and/or nitrate  b) pyuria (>10 white blood cells/mm3 or >3 white blood cells/high‐power field of unspun  urine)  c) organisms seen in Gram stain of unspun urine  d) frank pus expressed around the urinary catheter  e) at least two urine cultures with repeated isolation of the same uropathogen with ≥10²  colonies/ml in nonvoided specimens  f) urine culture with ≤ 105 colonies/mL of a single uropathogen in a patient being treated  with appropriate antimicrobial therapy  g) radiographic evidence of infection (e.g., ultrasound, computed tomography, magnetic  resonance imaging, radiolabeled scan) |
| Definite | 1. See criteria at possible  **and** urine culture with >105 colonies/mL with no more than two species of microorganisms  2. Abscess or other evidence of infection seen on direct examination, during surgery, or by  histopathologic examination |

| **Urosepsis in catheterized patients** | |
| --- | --- |
| Clinical  setting | Patients who have an urinary catheter or that has been removed within the past 6 days  presenting with symptoms of urinary tract infection |
| Possible | 1. At least two of the following signs or symptoms with no other recognized cause  a) Fever (>38 °C)  b) positive dipstick for leukocyte esterase and/or nitrate  c) pyuria (>10 white blood cells/mm3 or >3 white blood cells/high‐power field of unspun  urine)  d) organisms seen in Gram stain of unspun urine  e) frank pus expressed around the urinary catheter  f) two urine cultures with repeated isolation of the same uropathogen with ≥10²  colonies/ml in nonvoided urine (Gram negative pathogen or S. Saprophyticus)  g) urine culture with ≤ 105 colonies/mL of single uropathogens in a patient being treated  with appropriate antimicrobial therapy  h) hematuria |
| Probable | 1. See criteria at possible  **and** urine culture with >105 colonies/mL and no more than 2 species of pathogens |
| Definite | 1. See criteria at probable  **and** purulent drainage from the affected site (nefrostomy)  **and** organisms cultured from blood that are compatible with urine culture  2. Abscess or other evidence of infection seen on direct examination, during surgery, or by  histopathologic examination  **and** bacteremia |
| Comment | • Urine culture must be obtained using appropriate technique, such as clean catch collection or catheterization |

| **Upper urinary tract infection (kidney, ureter, bladder, urethra, or tissues surrounding the retroperitoneal or perinephric spaces)** | |
| --- | --- |
| Possible | 1. At least two of the following signs or symptoms with no other recognized cause  a) fever (>38 °C)  b) urgency  c) localized pain or tenderness at involved site |
| Probable | 1. See criteria at possible  **and** at least one of the following:  a) purulent drainage,  b) pyuria,  c) hematuria,  d) positive culture,  e) positive Gram stain  f) radiographic evidence of infection (e.g., ultrasound, computed tomography, magnetic  resonance imaging, radiolabeled scan)  **and** bacteremia |
| Definite | 1. Organism isolated from culture (other than urine) or tissue from the affected site  2. Abscess or other evidence of infection seen on direct examination, during surgery, or by  histopathologic examination |

**Skin and soft tissue**

| **Skin infection, erysipelas, phlebitis** | |
| --- | --- |
| Possible | 1. At least two of the following signs or symptoms with no other recognized cause:  a) pain or tenderness b) localized swelling  c) redness d) heat |
| Probable | 1. See criteria at possible  **and** organisms cultured from blood |
| Definite | 1. Purulent drainage, pustules, vesicles, or boils  2. See criteria at possible  **and** organisms cultured from aspirate or drainage of affected site; if organisms are normal  skin flora (e.g. coagulase‐negative staphylococci, micrococci, diphtheroids) they must be a  pure culture |

| **Surgical site infections: Superficial wounds** | |
| --- | --- |
| Clinical  setting | Patients presenting with symptoms or signs of wound infection within 30 days following surgery  or trauma |
| Possible | 1. Infection that arises within 30 days of an operative procedure and at the site of surgical  intervention  **and** infection involves the skin or subcutaneous tissue alone  **and** at least two of the following:  a) pain or tenderness b) localized swelling  c) redness d) heat |
| Probable | N.A. |
| Definite | 1. See criteria at possible  **and** at least one of the following:  a) purulent discharge from incision or drain  b) abscess seen during observation, (re)surgery, histopathologic or radiographic  examination  c) organisms cultured from tissue or drainage of affected site |
| Comment | • Infections around surgical sutures and infected burn wounds are not classified as superficial  POWI’s |

| **Surgical site infections: Deep wounds** | |
| --- | --- |
| Clinical  setting | Patients presenting with symptoms or signs of wound infection within 30 days following surgery  or trauma |
| Possible | 1. Infection that arises within 30 days of an operative procedure and at the site of surgical  intervention or within 1 year after implant placement  **and** Infection involves the fascia or muscle layers  **and** at least two of the following:  a) pain or tenderness b) localized swelling  c) redness d) heat |
| Probable | N.A. |
| Definite | 1. See criteria at possible  **and** at least one of the following:  a) purulent discharge from incision or drain  b) abscess seen during observation, (re)surgery, histopathologic or radiographic  examination  c) organisms cultured from tissue or drainage of affected site |

| **Soft tissue infections** | |
| --- | --- |
| Clinical  setting | Patients presenting with symptoms of infections of soft tissue such as necrotizing fasciitis,  infectious gangrene and necrotizing cellulitis and myositis, lymphadenitis and lymphangitis |
| Possible | 1. At least two of the following signs or symptoms with no other recognized cause:  a) pain or tenderness  b) localized swelling c) redness  d) heat |
| Probable | 1. See criteria at possible  **and** at least two of the following:  a) organisms cultured from blood;  b) signs suggestive for infection on computed tomography or magnetic resonance imaging |
| Definite | 1. Organisms cultured from tissue or drainage of affected site  2. Purulent drainage from affected site  3. Abscess or other evidence of infection seen during observation, surgery, histopathologic or radiographic examination |

| **Decubitus infections** | |
| --- | --- |
| Possible | 1. Clinical signs of infection (stage 3 or more) with the following signs or symptoms with no  other recognized cause:  a) redness  b) tenderness  c) swelling of decubitus wound edges |
| Probable | 1. See criteria at possible  **and** organisms cultured from blood and absence of other evidence of infections |
| Definite | 1. See criteria at possible  **and** organisms cultured from a decubitus ulcer by needle aspiration of fluid or biopsy of  tissue from the ulcer margin  **and** evident pus from wound |
| Comments | • Purulent drainage alone is not sufficient evidence of an infection  • Organisms cultured from the surface of a decubitus ulcer are not sufficient evidence that the ulcer is infected. A properly collected specimen from a decubitus ulcer involves needle aspiration of fluid or biopsy of tissue from the ulcer margin |

**Infections of the GI tract**

| **Intra‐abdominal infection/abscess (IAI)** | |
| --- | --- |
| Clinical  setting | Patients presenting with a localized pus collection in peritoneal cavity or nearby abdominal  structures in the absence of a documented gastrointestinal perforation |
| Possible | 1. At least two of the following signs or symptoms with no other recognized cause:  a) pain  b) diarrhea  c) fever  d) vomiting |
| Probable | 1. See criteria at possible  **and** radiographic and/or surgical evidence of abscess  **and** purulent drainage and/or positive Gram stain of drainage |
| Definite | 1. See criteria at probable  **and** organisms cultured from aspiration and/or blood |

| **Pancreatic infection, infected necrotizing pancreatitis** | |
| --- | --- |
| Possible | 1. Radiographic or direct surgical inspection with evidence suggestive of pancreatic abscess or  other type of infection |
| Probable | 1. The presence of surgical or radiographic evidence of an abnormal collection of an  inflammatory focus within the substance of the pancreas or surrounding structures  **and** a positive Gram stain from the pancreatic collection in the absence of culture documentation |
| Definite | 1. This requires direct confirmation of positive microbial cultures from the pancreas or  surrounding structures by percutaneous aspiration or direct visualization and culture at the time of surgery or from the bloodstream |

| **Biliary tract infection** | |
| --- | --- |
| Possible | 1. At least two of the following clinical symptoms of biliary tract infection:  a) pain right upper quadrant  b) fever  c) jaundice  d) colic pain |
| Probable | 1. See criteria at possible  **and** positive Gram stain from the biliary system  **and** radiographic evidence on ultrasound/CT |
| Definite | 1. See criteria at probable  **and** at least one of the following:  a) the isolation of pathogenic microorganisms obtained via percutaneous or direct surgical collection of samples in the lumen of the gall bladder or the biliary tract or the blood  b) ERCP with quick recovery  c) cholecystectomy with recovery |
| Comments | • A positive blood culture in a patient with ascending cholangitis is sufficient to diagnose a biliary tract infection  • A positive culture from the biliary tract in a patient without symptoms is insufficient to diagnose a biliary tract infection  • A positive culture from a T‐tube drain from the biliary tract is insufficient to diagnose a biliary tract infection when the tube is more than 24 hours in situ |

| **Primary peritonitis (spontaneous bacterial peritonitis)** | |
| --- | --- |
| Clinical  setting | Patients presenting with an infection of the peritoneal fluid in the absence of a gastrointestinal  perforation, abscess, or other localized infection within the gastrointestinal tract |
| Possible | 1. At least two of the following signs or symptoms with no other recognized cause:  a) fever  b) abdominal pain in more than 1 quadrant (not localized)  c) ileus  d) feeding intolerance  **and** inflammatory peritoneal fluid (>500 leucocytes/mL) in the absence of a positive culture  (in peritoneal fluid or blood) or Gram stain |
| Probable | 1. See criteria at possible  **and** evidence of an inflammatory ascitic fluid (>500 leukocytes/mL with a neutrophil predominance) in the presence of a positive Gram stain but negative peritoneal fluid cultures or in the presence of a positive blood culture for a pathologic organism with inflammatory cells in ascitic fluid |
| Definite | 1. Clinical signs of peritonitis  **and** isolation of microbial pathogens (in peritoneal fluid or blood) |

| **Secondary peritonitis** | |
| --- | --- |
| Clinical  setting | Patients presenting with an infection of the peritoneal space following perforation, abscess  formation, ischemic necrosis, or penetrating injury of the intra‐abdominal contents |
| Possible | 1. Upper gastrointestinal perforation or penetrating abdominal trauma that is surgically  repaired without further evidence of microbiologic confirmation or clinical signs or  symptoms supportive of a diagnosis of bacterial or fungal peritonitis  2. A finding of an inflammatory peritoneal fluid in the presence of a documented but localized  intra‐abdominal abscess in the absence of culture confirmation  3. At least two of the following signs or symptoms with no other recognized cause:  a) fever  b) abdominal pain in more than 1 quadrant (not localized)  c) ileus  d) feeding intolerance |
| Probable | 1. See clinical signs at possible  **and** at least one of the following:  a) Documented evidence of perforation (free air in the abdomen on radiographic studies or surgical confirmation of peritoneal inflammation following luminal perforation in the absence of microbiologically confirmed peritonitis)  b) Gram stain in the absence of a positive culture from the peritoneum |
| Definite | 1. See clinical signs at possible  **and** isolation of one or more microbial pathogens found in the peritoneum or the blood 24  hrs after a gastrointestinal perforation of the stomach, esophagus or duodenum, or any perforation of the small bowel distal to the ligament of Treitz |
| Comments | • Spillage of luminal contents during an operative procedure is not sufficient evidence of perforation that allows for definitive diagnosis of peritonitis  • Furthermore, a penetrating abdominal wound or documented perforation that is surgically repaired within 12 hrs of its occurrence is not sufficient evidence to support diagnosis of secondary bacterial peritonitis |

| **Tertiary peritonitis** | |
| --- | --- |
| Clinical  setting | Patients presenting with a persistent intra‐abdominal inflammation and clinical signs of  peritoneal irritation following secondary peritonitis from nosocomial pathogens |
| Possible | 1. Clinical signs of peritonitis (see secondary peritonitis)  **and** persistent signs of systemic inflammation but without clear documented evidence of  persistent inflammation within the peritoneal space following secondary bacterial peritonitis |
| Probable | 1. Clinical signs of peritonitis with documented secondary peritonitis  **and** persistent peritoneal inflammation (500 leukocytes/mL peritoneal fluid) in the absence  of microbiologically confirmed microbial persistence in the peritoneal space |
| Definite | 1. Isolation of one or more nosocomial pathogens from peritoneal fluid or blood in an  appropriate clinical situation (48 hrs after treatment for primary or secondary peritonitis) |

| **Peritoneal dialysis‐related peritonitis** | |
| --- | --- |
| Clinical  setting | Patients with peritoneal dialysis presenting with an infection of the peritoneal fluid |
| Possible | 1. Clinical signs of peritonitis in patients receiving peritoneal dialysis (see secondary peritonitis)  **and** abnormal accumulation of inflammatory cells in the peritoneum (100 leukocytes/mL)  but without absence of Gram strain and culture evidence of infection |
| Probable | 1. Clinical signs of peritonitis  **and** an inflammatory process (100 leukocytes/mL) of the peritoneum during the course of  peritoneal dialysis  **and** positive Gram stain  but without culture documentation from blood or the peritoneal space ) |
| Definite | 1. Clinical signs of peritonitis  **and** an inflammatory process (100 leukocytes/mL) of the peritoneum  **and** presence of culture documentation in peritoneal fluid or blood of a pathogenic  microorganism) |

| **Gastroenteritis** | |
| --- | --- |
| Possible | 1. Acute onset of diarrhea (liquid stools for more than 12 hours) of unknown cause (infectious  or non‐ infectious)  **and** at least 5 times a day of minimal 500 mL liquid stool (patients with laxatives are excluded in case no evidence of infection) |
| Probable | 1. Acute onset of diarrhea (liquid stools for more than 12 hours) and no likely noninfectious  cause |
| Definite | 1. At least two of the following symptoms:  a) nausea  b) vomiting  c) abdominal pain  d) headache  **and** enteric pathogen is cultured from stool or rectal swab |

**Infections of the reproductive system**

| **Endometritis, epididymitis, prostatitis** | |
| --- | --- |
| Possible | 1. Purulent drainage from the uterus  2. Male: at least one of the following a) fever  b) enlarged testicle / epididymis |
| Probable | 1. Purulent drainage from the uterus  **and** at least two of the following:  a) fever (>38°C),  b) abdominal pain,  c) uterine tenderness  2. Male: see criteria at possible  **and** purulent drainage from the urethra |
| Definite | 1. Organisms cultured from fluid or tissue from endometrium obtained during surgical  operation, by needle aspiration, or by brush biopsy  2. Male: see criteria at probable  **and** evidence for infection on ultrasound |

**Bones and joints**

| **Osteomyelitis** | |
| --- | --- |
| Possible | 1. At least two of the following signs or symptoms with no other recognized cause:  a) fever (>38°C)  b) localized swelling  c) tenderness d) heat  e) drainage at suspected site of bone infection |
| Probable | 1. See criteria at possible  **and** radiographic evidence of infection (CT, MRI) |
| Definite | 1. Organisms cultured from bone  2. Evidence of osteomyelitis on direct examination of the bone during a surgical operation or  histopathologic examination  3. See criteria at probable  **and** organisms cultured from blood |

**Infections of mouth, tongue or gums**

| **Infections of mouth, tongue or gums** | |
| --- | --- |
| Possible | 1. At least one of the following signs or symptoms:  a) abscess  b) ulceration  c) raised white patches on inflamed mucosa d) plaques on oral mucosa |
| Probable | 1. See criteria at possible  **and** at least one of the following:  a) organisms seen on Gram stain;  b) positive KOH (potassium hydroxide);  c) multinucleated giant cells seen on microscopic examination of mucosal scapings;  d) diagnostic single antibody titer (IgM) or fourfold increase in paired sera (IgG) for  pathogen |
| Definite | 1. Abscess or other evidence of oral cavity infection seen on direct examination, during a  surgical operation, or during a histopathologic examination  2. See criteria at probable  **and** organisms cultured from purulent material from tissues or oral cavity |

**Other definitions**

| **CPIS** | | |
| --- | --- | --- |
| Tracheal secretions | None or scant  Non‐purulent  Purulent | 0  1  2 |
| Infiltrates on chest  radiograph | Absent  Diffuse  Localized | 0  1  2 |
| Fever (°C): | >36.5 en < 38.4  > 38.4 en < 38.9  > 38.9 of < 36 | 0  1  2 |
| Leukocytosis (/mm3): | > 4000 and < 11,000  < 4000 or > 11,000  < 4000 or > 11,000 en > 500 band forms | 0  1  2 |
| PaO2/FIO2: | > 240 or ARDS  < 240 and no ARDS | 0  2 |
| Microbiology | Negative  Positive | 0  2 |
