# Supplementary material for: Transfusion of platelets, but not of red blood cells, is independently associated with nosocomial infections in the critically ill
Source: Ann Intensive Care. 2016 Jul 19;6:67. doi: 10.1186/s13613-016-0173-1 (PMC4951387; doi:10.1186/s13613-016-0173-1)

**Online supplement**

**Transfusion of fresh frozen plasma and platelets, but not of red blood cells, is associated with nosocomial bacterial infections in the critically ill**

Leo J. Engele, BSc^1^, Marleen Straat, MD^1^, Ingeborg H.M. van Rooijen^2^, Karen M.K. de Vooght, PharmD, PhD^4^, Olaf L. Cremer, MD, PhD^5^, Marcus J. Schultz, Lieuwe D.J. Bos, PhD^1^  and Nicole P. Juffermans, MD, PhD^1,3^ ; on behalf of the MARS ConsortiumOnline Supplement

**Supplemental methods**

Propensity score

The a-priori likelihood of receiving a blood transfusion differs between ICU patients. In this study, in which we investigate the association between transfusion and infection, we corrected for all confounders of infection and also for the a-priori likelihood of transfusion. Variables which may influence the risk of receiving a blood transfusion were added to a logistic regression model for all blood products (with blood transfusion as dependent variable) to calculate a propensity score. This results in a likelihood, expressed as a probability between 0 and 1, for receiving the specific type of blood transfusion. This likelihood can be transformed into a continuous variable by transformation, as done commonly in logistic regression. This variable can now be used a co-variate in the following models and expresses the risk of patient of receiving a certain type of transfusion, based on clinical data.

**Supplemental tables.**

**Table S1.1** Comparison of the combination of blood transfusion products in all transfused patients (n=1304)

| Transfusion products | RBC | Plasma | Platelets |
| --- | --- | --- | --- |
| RBC | 536 (41%) | 132 (10.1%) | 125 (9.5%) |
| Plasma | 132 (10.1%) | 14 (1.1%) | 16 (1.2%) |
| Platelets | 125 (9.5%) | 16 (1.2%) | 39 (3%) |
|  |  | | |
| Combination all three | 440 (34%) | | |

**Table S1.2** Comparison of the combination of blood transfusion products in transfused patients with an infection (n=288)

| Transfusion products | RBC | Plasma | Platelets |
| --- | --- | --- | --- |
| RBC | 97 (33.7%) | 29 (10.1%) | 24 (8.3%) |
| Plasma | 29 (10.1%) | 5 (1.7%) | 3 (1%) |
| Platelets | 24 (8.3%) | 3 (1%) | 8 (2.8%) |
|  |  | | |
| Combination all three | 122 (42.4%) | | |

**Table S1.3** Patient characteristics according to transfusion product.

| Transfusion product | any RBC  (n=1233) | any Plasma  (n=602) | any Platelets  (n=620) |
| --- | --- | --- | --- |
| Mean age, years ± SD | 60.1 ± 16.2 | 60.6 ± 16.8 | 59.2 ± 16.3 |
| APACHE IV score, median (IQR) | 81 (64-103) | 80 (62-103) | 81 (63-105) |
| Immunosuppressive medication, n(%) | 157 (13) | 61 (10) | 87 (14) |
| Trauma, n(%) | 98 (8) | 62 (10) | 50 (8) |
| Malignancy, n(%) | 238 (19) | 94 (16) | 124 (20) |
| Sepsis, n(%) | 606 (49) | 232 (39) | 256 (41) |
| Mechanical ventilation, n(%) | 1146 (93) | 575 (96) | 587 (95) |

**Red blood cell transfusion.**

**Table S2.1** Logistic regression with selection of most relevant transfusion characteristics

| Variables removed | AIC |  |
| --- | --- | --- |
| RBC >14 days | -0.56 |  |
| RBC >21 days | -1.99 |  |
|  |  |  |
| Variables in model | Regression coefficient | p-value |
| RBC transfusion | 0.717 | <0.001 |
| RBC units | 0.048 | <0.001 |
|  |  |  |

**Table S2.2** Logistic regression with correction for confounders. Selection of most relevant clinical characteristics.

| Variables removed | AIC |  |
| --- | --- | --- |
| Admission type | -1.85 |  |
| Immunosuppressive condition | -3.17 |  |
| Immunosuppressive medication | -4.98 |  |
| Trauma | -5.37 |  |
| Malignancy | -5.13 |  |
|  |  |  |
| Variables in model | Regression coefficient | p-value |
| RBC transfusion | 0.729 | <0.001 |
| RBC units | 0.045 | <0.001 |
| Mechanical ventilation | 0.746 | 0.002 |
| APACHE IV predicted length of stay | 0.138 | <0.001 |
|  |  |  |

**Table S2.3** Calculation of propensity score for RBC transfusion and addition of the propensity score to the model.

| Variables removed from propensity score | AIC | |  |
| --- | --- | --- | --- |
| none |  | |  |
|  |  | |  |
| Variables in propensity score | Regression coefficient | | p-value |
| Admission type | 0.445 | | <0.001 |
| Trauma | 0.148 | | 0.071 |
| Malignancy | 0.347 | | 0.001 |
| APACHE IV score | 0.020 | | <0.001 |
| Sepsis | 0.227 | | 0.006 |
| Variables | Odds ratio | 95% Confidence interval | p-value |
| Propensity score | 1.157 | 0.993 - 1.378 | 0.061 |
| RBC transfusion | 1.977 | 1.535 - 2.547 | < 0.001 |
| RBC units | 1.044 | 1.026 - 1.063 | < 0.001 |
| Mechanical ventilation | 2.034 | 1.266 - 3.270 | 0.003 |
| APACHE IV Predicted length of stay | 1.141 | 1.077 - 1.210 | < 0.001 |

**Table S2.4** Cox proportional hazard model with the previously selected variables.

| Variables | Hazard | 95% confidence interval | p-value |
| --- | --- | --- | --- |
| Propensity score | 1.162 | 1.004 - 1.345 | 0.044 |
| RBC transfusion | 1.143 | 0.906 - 1.442 | 0.259 |
| RBC units | 1.014 | 1.000 - 1.028 | 0.053 |
| Mechanical ventilation | 1.281 | 0.811 - 2.022 | 0.288 |
| APACHE IV Predicted length of stay | 0.982 | 0.931 - 1.036 | 0.504 |

**Fresh Frozen Plasma transfusion**

**Table S3.1** Logistic regression with selection of most relevant transfusion characteristics

| Variables removed | AIC |  |
| --- | --- | --- |
| none |  |  |
| Variables in model | Regression coefficient | p-value |
| FFP transfusion | 0.822 | <0.001 |
| FFP units | 0.031 | 0.005 |

**Table S3.2** Logistic regression with correction for confounders. Selection of most relevant clinical characteristics.

| Variables removed | AIC |  |
| --- | --- | --- |
| Immunosuppressive condition | -2.00 |  |
| Admission type | -3.97 |  |
| Malignancy | -5.45 |  |
| Immunosuppressive medication | -6.93 |  |
| Trauma | -7.38 |  |
| FFP units | -4.34 |  |
| Variables in model | Regression coefficient | p-value |
| FFP transfusion | 1.053 | <0.001 |
| Mechanical ventilation | 0.765 | 0.001 |
| APACHE IV predicted length of stay | 0.161 | <0.001 |

**Table S3.3** Calculation of propensity score for FFP transfusion and addition of the propensity score to the model.

| Variables removed from propensity score | AIC | |  |
| --- | --- | --- | --- |
| Malignancy | -1.58 | |  |
| Trauma | -0.65 | |  |
|  |  | |  |
| Variables in propensity score | Regression coefficient | | p-value |
| Admission type | 0.707 | | <0.001 |
| APACHE IV score | 0.017 | | <0.001 |
| Sepsis | -0.263 | | 0.011 |
| Variables | Odds ratio | 95% confidence interval | p-value |
| Propensity score | 1.294 | 1.120 - 1.494 | < 0.001 |
| FFP transfusion | 2.510 | 1.978 - 3.186 | < 0.001 |
| Mechanical ventilation | 1.950 | 1.214 - 3.132 | 0.006 |
| APACHE IV Predicted length of stay | 1.171 | 1.106 - 1.240 | < 0.001 |

**Table S3.4** Cox proportional hazard model with the previously selected variables.

| Variables | Hazard | 95% confidence interval | p-value |
| --- | --- | --- | --- |
| Propensity score | 1.347 | 1.180 - 1.537 | < 0.001 |
| FFP transfusion | 1.362 | 1.101 - 1.685 | 0.004 |
| Mechanical ventilation | 1.154 | 0.730 - 1.824 | 0.541 |
| APACHE IV Predicted length of stay | 0.996 | 0.945 - 1.050 | 0.881 |

**Platelet transfusion**

**Table S4.1** Logistic regression with selection of most relevant transfusion characteristics

| Variables removed | AIC |  |
| --- | --- | --- |
| PLT >3 days | -1.66 |  |
| Variables in model | Regression coefficient | p-value |
| PLT transfusion | 0.829 | <0.001 |
| PLT units | 0.045 | 0.026 |

**Table S4.2** Logistic regression with correction for confounders. Selection of most relevant clinical characteristics.

| Variables removed | AIC |  |
| --- | --- | --- |
| Admission type | -1.71 |  |
| Immunosuppressive medication | -3.26 |  |
| Immunosuppressive condition | -4.94 |  |
| Malignancy | -4.73 |  |
| PLT units | -4.83 |  |
| Trauma | -4.22 |  |
| Variables in model | Regression coefficient | p-value |
| PLT transfusion | 1.054 | <0.001 |
| Mechanical ventilation | 0.786 | 0.001 |
| APACHE IV predicted length of stay | 0.163 | <0.001 |

**Table S4.3** Calculation of propensity score for platelet transfusion and addition of the propensity score to the model.

| Variables removed | AIC | |  |
| --- | --- | --- | --- |
| Trauma | -1,92 | |  |
| Variables in propensity score | Regression coefficient | | p-value |
| Malignancy | 0.433 | | <0.001 |
| Admission type | 0.593 | | <0.001 |
| APACHE IV score | 0.019 | | <0.001 |
| Sepsis | -0,248 | | 0.015 |
| Variables | Odds ratio | 95% confidence interval | p-value |
| Propensity score | 1.308 | 1.126 - 1.520 | < 0.001 |
| PLT transfusion | 2.530 | 1.998 - 3.205 | < 0.001 |
| Mechanical ventilation | 1.984 | 1.235 - 3.187 | 0.005 |
| APACHE IV Predicted length of stay | 1.174 | 1.109 - 1.244 | < 0.001 |

**Table S4.4** Cox proportional hazard model with the previously selected variables.

| Variables | Hazard | 95% confidence interval | p-value |
| --- | --- | --- | --- |
| Propensity score | 1.297 | 1.130 - 1.489 | < 0.001 |
| PLT transfusion | 1.463 | 1.184 - 1.806 | < 0.001 |
| Mechanical ventilation | 1.205 | 0.763 - 1.904 | 0.423 |
| APACHE IV Predicted length of stay | 1.000 | 0.948 - 1.054 | 0.989 |

|  |  |
| --- | --- |

**Table S5** Logistic and cox regression model without pre-ICU blood transfusions for each type of transfusion separately.

|  | | | |  |  |  |  |
| --- | --- | --- | --- | --- | --- | --- | --- |
|  |  | **Logistic regression** |  |  |  | **Cox regression** |  |
| **Blood products** | Odds ratio | 95% CI | p-value |  | Hazard | 95% CI | p-value |
| **Red blood cells** |  |  |  |  |  |  |  |
| RBC transfusionᵃ | 2.080 | 1.620 - 2.670 | <0.001 |  | 1.160 | 0.922 - 1.459 | 0.210 |
| RBC Unitsᵃ | 1.041 | 1.022 - 1.060 | <0.001 |  | 1.010 | 0.996 - 1.025 | 0.167 |
| **Fresh Frozen Plasma** | |  |  |  |  |  |  |
| FFP transfusionᵇ | 2.542 | 1.990 - 3.191 | <0.001 |  | 1.420 | 1.143 - 1.764 | 0.002 |
| **Platelets** |  |  |  |  |  |  |  |
| Platelet transfusion ͨ | 1.610 | 2.131 - 3.168 | <0.001 |  | 1.561 | 1.260 - 1.933 | <0.001 |
| ᵃCorrected for exposure bias summarized in propensity score including trauma, admission type, sepsis, malignancy and APACHE IV score. And corrected for confounders including | | | | | | |  |
| APACHE predicted length of stay and mechanical ventilation. | | | | | | | |
| ᵇ Corrected for exposure bias summarized in propensity score including admission type, APACHE IV score and sepsis. And corrected for confounders including APACHE predicted length of stay and mechanical ventilation. | | | | | | | |
| ͨ Corrected for exposure bias summarized in propensity score including admission type, sepsis, malignancy and APACHE IV score. And corrected for confounders including APACHE predicted | | | | | | | |
| length of stay and mechanical ventilation. | | | | | | | |
| Abbreviations: FFP: fresh frozen plasma, RBC: red blood cells | | | | | | | |

**Table S6** Analysis of independent transfusion risk factors for nosocomial infection without pre-ICU blood transfusions

|  | | | |  | |
| --- | --- | --- | --- | --- | --- |
|  |  | **Cox regression** |  |  | |
| **Blood products** | Hazard ratio | 95% CI | p-value |  | |
| **Red blood cells** |  |  |  |  | |
| RBC transfusion | 1.114 | 0.867 - 1.431 | 0.398 |  | |
| RBC Units | 0.991 | 0.973 - 1.010 | 0.340 |  | |
| **Fresh Frozen Plasma** | |  |  |  | |
| FFP transfusion | 1.092 | 0.806 – 1.480 | 0.569 |  | |
| **Platelets** |  |  |  |  | |
| Platelet transfusion | 1.482 | 1.098 – 2.002 | 0.030 |  | |
| Corrected for exposure bias summarized in propensity scores of the individual transfusion products and corrected for confounders including APACHE predicted length of stay and mechanical ventilation. | | | |  | |
| Abbreviations: FFP: fresh frozen plasma, RBC: red blood cells | | | |  | |
|  | | | |  | |
| Table S7. **Analysis of independent transfusion risk factors for nosocomial Infection with SOFA score on day 3.** | | | |  |  |
|  |  | **Cox regression** |  |  |  |
| **Blood products** | Hazard ratio | 95% CI | p-value |  |  |
| **Red blood cells** |  |  |  |  |  |
| RBC transfusion | 0.982 | 0.771 - 1.251 | 0.883 |  |  |
| RBC Units | 0.996 | 0.979 - 1.012 | 0.606 |  |  |
| **Fresh Frozen Plasma** | |  |  |  |  |
| FFP transfusion | 1.122 | 0.831 – 1.515 | 0.452 |  |  |
| **Platelets** |  |  |  |  |  |
| Platelet transfusion | 1.323 | 0.977 – 1.792 | 0.043 |  |  |
| *Corrected for exposure bias summarized in propensity scores of the individual transfusion products and corrected for confounders including SOFA score, trauma and mechanical ventilation. | | | |  |  |
| Abbreviations: FFP: fresh frozen plasma, RBC: red blood cells | | | |  |  |

Supplemental figures

**Figure S1** Schematic representation of the statistical analyses.


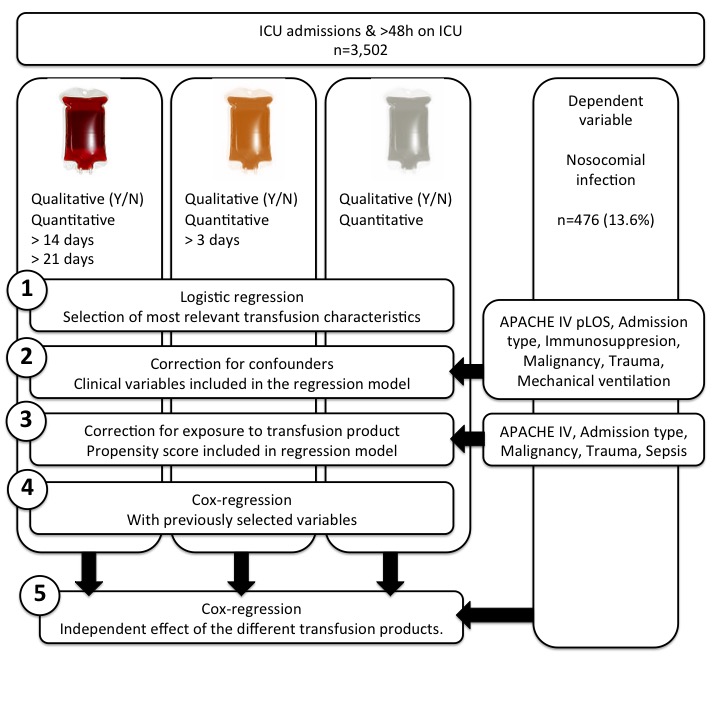

Supplement: Supplementary file 2 — 10.1186/s13613-016-0173-1 Supplemental statistical methods. Table S1.1 Comparison of the combination of blood transfusion products in all transfused patients. Table S1.2 2 Comparison of the combination of blood transfusion products in transfused patients with an infection. Table S1.3 Patient characteristics according to transfusion product. Tables S2. Models for RBC transfusion. Tables S3. Models of FFP transfusion. Table S4. Models for PLT transfusion. Table S5. Logistic and cox regression model for each type of transfusion separately. Table S6 Analysis of independent transfusion risk factors for nosocomial infection. Table S7. Analysis of independent transfusion risk factors for nosocomial Infection with SOFA score on day 3. [file 13613_2016_173_MOESM2_ESM.docx]
